# Brain structure and allelic associations in Alzheimer's disease

**DOI:** 10.1111/cns.14073

**Published:** 2022-12-27

**Authors:** Seok Woo Moon, Lu Zhao, William Matloff, Sam Hobel, Ryan Berger, Daehong Kwon, Jaebum Kim, Arthur W. Toga, Ivo D. Dinov, for the Alzheimer's Disease Neuroimaging Initiative

**Affiliations:** ^1^ Department of Neuropsychiatry, Research Institute of Medical Science Konkuk University School of Medicine Seoul Korea; ^2^ USC Stevens Neuroimaging and Informatics Institute, Keck School of Medicine of USC California Los Angeles USA; ^3^ Microbiology & Immunology University of Michigan Ann Arbor Michigan USA; ^4^ Department of Biomedical Science and Engineering Konkuk University Seoul Korea; ^5^ Department of Health Behavior and Biological Sciences, Statistics Online Computational Resource (SOCR), Michigan Institute for Data Science (MIDAS) University of Michigan Ann Arbor Michigan USA; ^6^ Department of Statistics University of California Los Angeles California USA

**Keywords:** ADNI, Alzheimer's disease, genetics, mild cognitive impairment, networking, neuroimaging

## Abstract

**Background:**

Alzheimer's disease (AD), the most prevalent form of dementia, affects 6.5 million Americans and over 50 million people globally. Clinical, genetic, and phenotypic studies of dementia provide some insights of the observed progressive neurodegenerative processes, however, the mechanisms underlying AD onset remain enigmatic.

**Aims:**

This paper examines late‐onset dementia‐related cognitive impairment utilizing neuroimaging‐genetics biomarker associations.

**Materials and Methods:**

The participants, ages 65–85, included 266 healthy controls (HC), 572 volunteers with mild cognitive impairment (MCI), and 188 Alzheimer's disease (AD) patients. Genotype dosage data for AD‐associated single nucleotide polymorphisms (SNPs) were extracted from the imputed ADNI genetics archive using sample‐major additive coding. Such 29 SNPs were selected, representing a subset of independent SNPs reported to be highly associated with AD in a recent AD meta‐GWAS study by Jansen and colleagues*.*

**Results:**

We identified the significant correlations between the 29 genomic markers (GMs) and the 200 neuroimaging markers (NIMs). The odds ratios and relative risks for AD and MCI (relative to HC) were predicted using multinomial linear models.

**Discussion:**

In the HC and MCI cohorts, mainly cortical thickness measures were associated with GMs, whereas the AD cohort exhibited different GM‐NIM relations. Network patterns within the HC and AD groups were distinct in cortical thickness, volume, and proportion of White to Gray Matter (pct), but not in the MCI cohort. Multinomial linear models of clinical diagnosis showed precisely the specific NIMs and GMs that were most impactful in discriminating between AD and HC, and between MCI and HC.

**Conclusion:**

This study suggests that advanced analytics provide mechanisms for exploring the interrelations between morphometric indicators and GMs. The findings may facilitate further clinical investigations of phenotypic associations that support deep systematic understanding of AD pathogenesis.

## Graphical abstract

Heterogeneous, genetic, clinical, and neuroimaging data were used for model‐based inference and network analysis. An end‐to‐end data analysis workflow protocol to identify the most significant associations between neuroimaging, SNP, and clinical diagnosis biomarkers.

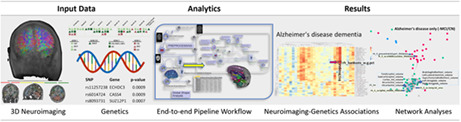

## 1 INTRODUCTION

Alzheimer's disease (AD) is by far the most common form of dementia among the elderly.^1^, ^2^ Late onset Alzheimer's disease (LOAD), defined by the onset of symptoms after age 65, is sporadic, non‐familial AD.^3^, ^4^ Genetic studies have provided significant insights on the molecular basis of AD, but the mechanisms underlying AD onset and progression remain largely unexplained. While the underlying causes of LOAD are still unknown, there is evidence from familial aggregation, transmission pattern, and twin studies that AD has a substantial genetic component that has an estimated heritability of 58%–79%, and the lifetime risk of AD among first‐degree relatives of patients may be twice that of the general population.^5^, ^6^ Recent genome‐wide association studies (GWASs), which examine associations of AD diagnosis with genetic markers (single‐nucleotide polymorphism [SNP]) across the genome, have discovered more than 20 AD gene variants that confer genetic risk.^7^, ^8^ These findings improve the understanding of risks and causes for AD, and may guide diagnosis and therapy on a patient‐specific basis.^9^ However, case–control GWAS cannot completely characterize the exact roles of the identified genetic susceptibility loci in the pathophysiology of AD. Joint analysis of genetic and neuroimaging data could uncover the genetic mechanism in the disease's underlying biology.^5^

This study investigates holistically the significance of multi‐gene patterns associated with neuroimaging markers (NIMs) of AD using imaging and genomic data of the Alzheimer's Disease Neuroimaging Initiative (ADNI) cohort.^10^ Several single‐nucleotide polymorphisms (SNPs) present in apolipoprotein E (*APOE*) gene have previously been associated with neuroimaging measures in both cognitively healthy control (HC) or impaired (such as mild cognitive impairment [MCI] and AD dementia) patients.^11^, ^12^, ^13^ However, a single gene or a few imaging measures may be insufficient to understand the multiple mechanisms and imaging manifestations of the complex disease. Recent and ongoing advances in neuroimaging and genetics, including high‐throughput genotyping techniques, have made it possible to scan populations with multimodality neuroimaging to collect genome‐wide data and to study the influence of genetic variation on the brain structure and function.

In this work, we related high‐throughput neuroimaging‐derived phenotypes of brain structure to the clinical states of AD, and then associated the significant AD‐related NIMs with GWAS‐supported susceptibility genetic variants for AD to obtain true system‐level gene‐brain associations in dementia. Specifically, we used structural brain imaging to obtain biomarkers of a wide variety of brain morphological properties, allelic data to capture genotypic variation, and functional connectivity to evaluate imaging‐genetic‐phenotypic variation. We present a neuroimaging genetics framework that uses a whole‐genome‐and‐whole‐brain strategy to systematically evaluate genetic effects on neuroimaging phenotypes to discover quantitative trait (QT) loci. QT association studies have been shown to have increased statistical power and thus decreased sample size requirements.^14^, ^15^ In addition, neuroimaging phenotypes may be closer to the underlying biological etiology of the disease, making it easier to identify underlying genes. The methodology proposed in this paper is based on the identification of strong associations between regional neuroimaging phenotypes as QTs and SNP genotypes as QT loci.

The genetics of AD are complex because the practical effects may be weak, albeit statistical effects could still be strong, sample‐sizes are often unbalanced (number of cases ≪ genomic markers [GMs]), and considerable difficulties with result replication and validation.^16^, ^17^ Large‐scale GWAS shows promise in untangling the genetic footprint of this neurodegenerative disease. Considering the limited sample size in the ADNI cohort (*n* = ~1200), we used the AD‐related genetic variants identified by the largest (*n* = ~450,000) case–control GWAS in AD to date^8^ instead of performing GWAS on the ADNI cohort.

We hypothesized that there exist significant relationships between the AD‐related NIMs and the GWAS‐supported susceptibility genetic variants for AD. Several prior studies have been conducted on the relationship between neuroimaging phenotypes and genetic variants.^18^, ^19^, ^20^ However, few reports have previously performed functional analyses of neuroimaging genetics. This study expands the knowledge about dementia phenotypes using modern neuroimaging genetics and the network analysis to explore the relationships between genetic, phenotypic, and NIMs.

## 2 METHODS

### 2.1 Inclusion–Exclusion criteria

Data used in the preparation of this article were obtained from the Alzheimer's Disease Neuroimaging Initiative (ADNI) database (https://adni.loni.usc.edu). ADNI was launched in 2003 as a public‐private partnership, led by Principal Investigator Michael W. Weiner. The primary goal of ADNI has been to test whether serial magnetic resonance imaging (MRI), positron emission tomography (PET), other biological markers, and clinical and neuropsychological assessment can be combined to measure the progression of mild cognitive impairment (MCI) and early Alzheimer's disease (AD). For up‐to‐date information, see https://www.adni‐info.org.

This study is based on participants from ADNI‐1, ADNI‐GO, and ADNI‐2. Only data from the baseline visit of each participant was considered. Each baseline participant has multiple structural MRI scans available with varying scan parameters (e.g., accelerated vs. non‐accelerated) and at various stages of preprocessing. Image selection aimed to maximize consistency across participants. First, the maximally preprocessed T1‐weighted MRI scans were downloaded for each participant.

For ADNI‐1 participants, quality assessment data available on the LONI IDA^21^ (MRI MPRAGE Ranking: MRIMPRANK) was used to select the higher‐rated scan for each visit. Accelerated scans were removed and 3T scans were chosen over 1.5T scans where possible. For those with multiple scans after this filtering, the scan with the larger Image ID was kept. For the ADNI‐GO and ADNI‐2 participants, accelerated scans were removed and among the remaining participants with more than one scan, the scan with the larger Image ID was kept. After these filtering steps were completed, the scans were combined into a final set of scans consisting of 1242 participants with only one scan each. The ADNI database query yielded a pool of 1242 volunteers with both brain MRI and genetic data from the study phases of ADNI‐1, ADNI‐GO and ADNI‐2. Three subjects were discarded due to failed MRI processing. We further removed 110 participants who did not have CEU ancestry (*ancestry from Northern and Western Europe*) and employed the remaining 1129 subjects for identifying the AD‐related NIMs. In the final imaging‐genetics association analysis another 103 subjects were excluded as they did not pass the genetic data quality control. We ended up with 1026 subjects who successfully completed all imaging and genetics processing protocols. For each participant, clinical severity of dementia was assessed using an annual semi‐structured interview, which yielded an overall Clinical Dementia Rating (CDR) score and the CDR Sum of Boxes (CDR‐SB). In addition, the Mini‐Mental State Examination (MMSE) and a neuropsychological battery were also recorded.

All the ADNI participants included in this study were those with a baseline age of 65–85 years, a baseline MMSE score of 20–30, and available genetic and imaging data. The 1026 ADNI participants included: 266 HC's (CDR = 0, Male:138, Female:128), 572 MCI's (CDR = 0.5, Male:227, Female:245), and 188 AD dementia patients (CDR = 0.5/1, Male:102, Female:86). We analyzed NIMs among participants with different APOE haplotype in the individual HC, MCI, and AD dementia cohorts. Subjects with AD dementia were probable AD dementia according to the NIA‐AA diagnostic criteria for AD. The ADNI study‐design specified that the time period between initial screening and clinical exams and the subsequent MRI imaging is about 3 months.

### 2.2 MRI processing and analysis

The MRI acquisition protocols can be found on the ADNI website (https://adni.loni.usc.edu) and have been previously described elsewhere.^22^, ^23^ Baseline structural MRI scans of the ADNI subjects were processed for reconstructing cortical surfaces, brain parcellation and extracting morphological phenotypes using the FreeSurfer (v6.0) software package (https://surfer.nmr.mgh.harvard.edu/).^24^ The FreeSurfer processing includes motion correction and averaging of volumetric T1‐weighted images,^25^ removal of non‐brain tissue,^26^ automated Talairach transformation, brain volume segmentation,^27^, ^28^ intensity normalization,^29^ tessellation of the boundary between gray matter and white matter, automated topology correction^30^ and surface deformation.^31^

Once the cortical models are completed, a number of deformable procedures were performed for further data processing and analysis, including surface inflation, registration to a spherical atlas using individual cortical folding patterns to match cortical geometry across subjects,^32^ and finally creation of a variety of surface‐based data including maps of surface area, cortical thickness, curvature features, etc.

For each subject, 1380 imaging‐derived biomarkers were extracted using FreeSurfer, including measures of surface area, volume, thickness, standard deviation of thickness, mean curvature, Gaussian curvature, folding index, curvature index and/or gray matter/white matter contrast for different cortical, subcortical, and white matter regions.

All the imaging phenotypes were adjusted for age, gender, education, handedness, and intracranial volume (ICV) using linear mixed‐effects regressions. ANOVA tests were then performed to find the NIMs associated with AD diagnosis at the GWAS significance level *p* < 5 × 10^−8^ in the cohort of *N* = 1129 subjects with a CEU ancestry.

### 2.3 Genetics data processing

Genetic SNP data was downloaded from the ADNI database (https://www.loni.usc.edu/ADNI) through the LONI imaging data archive (IDA) interface (https://ida.loni.usc.edu/pages/access/geneticData.jsp) onto the LONI Cranium high‐performance computing (HPC) cluster. The processing resulted in a single dataset containing the genetics information of all 1026 participants. The ADNI‐1 genetics data was downloaded as PLINK bed/bim/bam files in the hg18 (build 36) format. The genome build was converted from hg18 to hg19 using liftOver, as described in (https://www.nature.com/articles/nprot.2015.077). The ADNI‐GO and ADNI‐2 genetics data, which is in the hg19 (build 37) format, was downloaded as PLINK bed/bim/bam files for sets 1–9 and as intensity data CSV files for sets 10–15. The intensity data CSV files were converted to PLINK files at a multiple GenCall Score (GC) threshold of 0.15 based on the procedure described in Ref. [13].

ADNI‐1 and ADNI‐GO/2 used different genotyping chips. Used genetic imputation, we harmonized the genetic data across the different ADNI studies. To prepare for imputation, population stratification analysis was first used to remove all of the non‐CEU participants. We used PLINK for population stratification. PLINK relies on genome‐wide average proportion of alleles shared between any two individuals to cluster subjects into homogeneous subsets and perform classical multidimensional scaling (MDS) to visualize substructure and provide quantitative indices of population genetic variation. Next, we used the “HRC or 1000G Imputation preparation and checking” tool (HRC‐1000G‐check‐bim‐v4.2.9) from the McCarthy Group to conduct common pre‐imputation checks, such as strand, reference allele assignment, and frequency differences (https://www.well.ox.ac.uk/~wrayner/tools).

Imputation was completed using the Michigan Imputation Server v1.0.4 (Sept. 14, 2018) (https://imputationserver.sph.umich.edu/index.html). This imputation, based on Minimac3,^33^ was completed with the accompanying quality control offered by the service. The reference panel used was the HRC r1.1 2016, phasing was competing using Eagle, and quality control was based on the European population. The ADNI‐1 input consisted of 694 samples and 568,933 SNPs, 10,583 of which were excluded for imputation due to being monomorphic or having a SNP call rate of <90%. The ADNI‐GO/2 input consisted of 723 samples and 696,245 SNPs, 38,605 of which were excluded due to being monomorphic or having a SNP call rate of <90%. The output of the Michigan Imputation Server was in the minimac3 output format, including both info and dosage files. The HRC‐imputed data for the ADNI‐1 and ADNI‐GO/2 datasets were merged with *bcftools* (https://samtools.github.io/bcftools/bcftools.html). After this imputation and combination, the sites were filtered to only include those with *R* ^2^ > 0.6 and with a minor allele frequency >0.5% using *bcftools* and *tabix* (https://samtools.github.io/bcftools/bcftools.html, http://www.htslib.org). Finally, the data was converted into the *pgen* format (PLINK 2 binary format) using PLINK 2.00 alpha (https://www.cog‐genomics.org/plink/2.0/).

### 2.4 The pipeline computational environment

To manage the large and complex raw and derived data, design and execute the end‐to‐end processing protocols, and to track provenance, we employed the LONI Pipeline.^34^, ^35^, ^36^, ^37^ The Pipeline is a graphical workflow environment facilitating the collaborative design, execution, validation, visualization, modification and sharing of complex heterogeneous computational protocols.

To promote reproducible open‐science development and validation, we designed a Pipeline workflow that represents an end‐to‐end computational protocol for high‐throughput data preprocessing. The pipeline workflow includes skull‐stripping, volumetric registration, brain anatomical parcellation, extraction of volume and cortical thickness and between group statistical analyses of shape regional differences. The output of the pipeline workflow is a collection of 3D scenes illustrating the statistically significant regional anatomical differences between the study cohorts.

Rank‐ordering the complete collection of NIMs, we chose the 200 most salient NIMs which provided the highest discrimination between the AD and HC groups. These 200 NIMs were derived from all structural imaging data using the workflow and are based on the automated ROI extractions generated by FreeSurfer. Finally, the pipeline workflow, computed the most significant genotypic discriminants among AD, MCI and HC subjects. The 200 NIMs were then associated with the top 29 SNPs, which were chosen by the PLINK.^8^

### 2.5 Analytical protocol

The end‐to‐end data analysis protocol was implemented via the Pipeline graphical workflow environment and involved the following steps (1) Imputation on the Michigan Server using IMPUTE2, a genotype imputation and haplotype phasing program based on ideas from,^38^ and (2) Beagle is a software package for phasing genotypes and for imputing ungenotyped markers.^39^ The SOCR statistical computing infrastructure (https://SOCR.umich.edu)^40^, ^41^, ^42^, ^43^, ^44^, ^45^ was utilized to implement and execute the end‐to‐end computational statistics protocol, which included multivariate linear modeling and general parametric and non‐parametric statistical analyses.

The imputation protocol relied on the default setting. Following quality control (QC) and imputation, there were 1026 subjects with a European ancestry remaining with minimac3 outputs, including both info and dosage files. Next, we combined the ADNI‐1 and ADNI‐GO/2 imputed data into a single PLINK file. ADNI1 and ADNIGO/2 are genotyped using different chips, so we imputed the arrays prior to their integration and ran GWAS on the combined array. The resulting data were filtered to include only the subjects with a CEU ancestry and contained the following four tensors:

1. genetic.markers.associated.with.ADdx.CEU: included the SNPs, chromosomes, and positions corresponding to each SNP reference sequence ID, see Table 2. We replaced the *APOE* SNP (X19.45351516) with the separate *APOE* genotype test conducted by ADNI giving the exact *APOE* allele.
2. ADNI_baseline_CEU_metadata: contains the APOE genotype meta‐data.
3. imaging.markers.associated.with.ADdx.ANOVA: includes 200 NIMs and their corresponding association with clinical phenotypes (*p*‐values).
4. FS.stats.imaging.markers.associated.with.ADdx.CEU: contains the FreeSurfer‐extracted neuroimaging volume, surface and thickness measures for cortical and subcortical regions of interest (ROIs).

We extracted the AD‐related GMs and NIMs for the network analysis. Genotype dosage data for AD‐associated SNPs were extracted from the imputed ADNI genetics dosage data using sample‐major additive (0/1/2) coding. A set of 29 SNPs were selected, representing a subset of independent SNPs found to be highly associated with AD in a recent AD meta‐GWAS study by Jansen et al.,^8^ which met the MAF threshold determined by the imputation process. The genotyping data of these SNPs are included in the genetic.markers.associated.with.ADdx.CEU data object along with SNP IDs,^8^ Table 1. We replaced the *APOE* SNP (X19.45351516) with the separate *APOE* genotype test conducted by ADNI giving the exact *APOE* allele.

**TABLE 1:** Participants demographic data

Category	HC	MCI	AD	*p*‐Value
No. of subjects (1026)	266	572	188	
Gender (M/F)	138/128	227/245	102/86	0.580 (*)
Age	73.51 ± 3.89	70.74 ± 6.07	71.72 ± 6.34	<0.001 (*)
MMSE	29.07 ± 1.94	27.05 ± 1.79	27.75 ± 1.78	<0.0001 (**)
ADAS‐Cog	6.15 ± 2.86	11.43 ± 4.40	18.46 ± 6.28	<0.0001 (*)
Education (years, mean ± SD)	16.18 ± 2.68	15.98 ± 2.78	15.40 ± 2.87	0.010 (*)
Handedness (R/L)	207/18	348/35	188/12	0.418 (*)
APOE (ε2/ε3/ε4)	34/421/77	39/709/396	9/184/183	<0.0001 (**)

*Note*: *p*‐Values correspond to the appropriate chi‐square (*) or ANOVA (**) test statistics.

Abbreviations: AD, Alzheimer's disease; HC, healthy controls; MCI, mild cognitive impairment; MMSE, Mini‐Mental State Examination.

Age and gender were included as confounding factors in the analysis. The top 200 NIMs were identified to be associated with AD diagnosis at the GWAS significance level (*p* < 5 × 10^−8^). The 200 NIMs and their *p*‐values are stored in imaging.markers.associated.with.ADdx.ANOVA tensor. These NIMs were extracted and used to generate association heatmaps (SNPs by diagnosis and Imaging biomarker by diagnosis).

### 2.6 Neuroimaging‐genetics association analytics protocol

More details are provided in the Appendix S1 (Methods).

### 2.7 Network analysis between 29 genomic and 200 neuroimaging markers

The WGCNA (Weighted graph correlation network analysis) R package (version 1.68)^8^ was used to perform network analysis using 29 genomic and 200 neuroimaging markers. The aim of this analysis was to connect the NIMs having similar patterns observed from the GMs. WGCNA was originally developed to find the network of co‐expressed genes based on their expression patterns in multiple conditions. Specifically, for each of three cohorts, such as AD, MCI, and HC, the values of the NIMs and GMs were measured, and correlation coefficient values between the two kinds of markers were calculated. The correlation coefficient values constitute the correlation coefficient matrix (CCM) between the two kinds of markers. The CCM was then converted to matrices representing an unsigned adjacency matrix (using soft thresholding of 7) and a topology overlap matrix (using a score threshold of 0.05). The NIMs with similar genetic patterns were predicted by WGCNA using the converted matrices. The predicted networks for each of the AD, MCI, and HC cohort were visualized using Cytoscape (version 3.7.1).^46^

### 2.8 Statistically significant MCI/HC and AD/HC odds ratios

Using multinomial linear modeling of diagnosis, we studied the associations between the three individual cohorts (HC, MCI and AD dementia). The differences of the 200 NIMs and 29 SNPs between HC, MCI, and AD dementia cohorts. The results of a 3‐way ANOVA (ROI, Dx, SNP) may be less interpretable compared to a multinomial linear modeling (Outcome = Dx). We computed the odds ratios (ORs) and relative risks (RRs) for AD and MCI, relative to HC.

The MCI and AD effects quantified the metrics “relative to HC.” These represent extensions of the binary outcome in logistic regression, but reflect 3 categorical outcomes (HC, MCI, AD), which may also be analyzed via more general multi‐nominal linear modeling. In general, to assess statistical significance a customary false‐positive rate of *α* = 0.05 may be used for many different tests. However, in many GWAS studies, it's common that a correction for multiple comparison (e.g., false discovery rate, family wise error rate), or other strategies are used to control the false positive rate of significance.^47^, ^48^

## 3 RESULTS

### 3.1 Demographic characteristics

The demographics and clinical data of the ADNI participants at the baseline are summarized in Table 1 and include *p*‐values computed using Chi‐square or ANOVA, as appropriate. The 1,026 subjects (aged 65 ~ 85 years) were chosen from the ADNI datasets. The AD, MCI and HC subjects had statistically significant differences in age, MMSE, ADAS‐Cog, Education and APOE genotype.

### 3.2 AD genetic and imaging markers for the network analysis

We extracted the AD‐related genotypes and NIMs for the network analysis. Genotype dosage data for AD‐associated SNPs were extracted from the imputed ADNI genetics dosage data using sample‐major additive (0/1/2) coding. The genotypes, SNPs, chromosomes, and positions can be found in^8^ Table 2.

**TABLE 2:** Twenty nine genomics markers

[Top 29 genomics markers^8^]			
Index	GM (ADNI)	SNPs	Genes
1	X1.161155392_G	rs4575098	*[ADAMTS4]*
2	X1.207786828_A	rs2093760	*[CR1]*
3	X2.127891427_A	rs4663105	*[BIN1]*
4	X2.233981912_G	rs10933431	*[INPP5D]*
5	X4.11026028_A	rs6448453	*[CLNK]*
6	X4.11723235_A	rs7657553	*[HS3ST1]*
7	X6.32583357_A	rs6931277	*[HLA‐DRB1]*
8	X6.47432637_C	rs9381563	*[CD2AP]*
9	X7.99971834_A	rs1859788	*[ZCWPW1]*
10	X7.143108158_T	rs11763230	*[EPHA1]*
11	X8.27464929_A	rs4236673	*[CLU/PTK2B]*
12	X10.11717397_T	rs11257238	*[ECHDC3]*
13	X11.59958380_C	rs2081545	*[MS4A6A]*
14	X11.85776544_G	rs867611	*[PICALM]*
15	X11.121435587_T	rs11218343	*[SORL1]*
16	X14.92938855_G	rs12590654	*[SLC24A4]*
17	X15.59022615_T	rs442495	*[ADAM10]*
18	X15.63569902_C	rs117618017	*[APH1B]*
19	X16.31133100_G	rs59735493	*[KAT8]*
20	X17.5138980_G	rs113260531	*[SCIMP]*
21	X17.47450775_G	rs28394864	*[ABI3]*
22	X17.56409089_G	rs2632516	*[BZRAP1‐AS1]*
23	X18.29088958_C	rs8093731	*[SUZ12P1]*
24	X18.56189459_T	rs76726049	*[ALPK2]*
25	X19.1039323_C	rs111278892	*[ABCA7]*
26	X19.45351516_C	rs41289512	*[APOE]*
27	X19.46241841_C	rs76320948	*[AC074212.3]*
28	X19.51727962_C	rs3865444	*[CD33]*
*29*	*X20.54998544_A*	*rs6014724*	*[CASS4]*

Abbreviation: SNP, single‐nucleotide polymorphism.

To streamline the analyses, we chose the top 200 NIMs corresponding to the lowest *p*‐values for the discrimination between HC and AD subjects to correlate with 29 GMs, Table S1.

### 3.3 Association analysis among genetic and neuroimaging biomarkers

**FIGURE 1:**
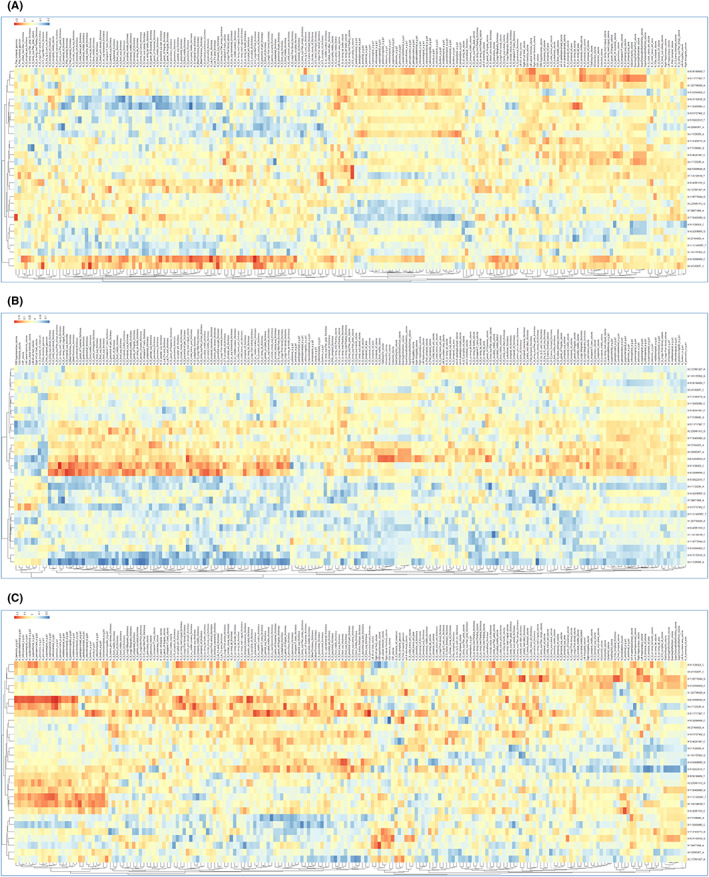
Dendrograms illustrating Neuroimaging‐Genetics Associations. (A) Cognitively asymptomatic control (HC) cohort; (B) Mild Cognitive Impairment (MCI) cohort; (C) Alzheimer's Disease (AD) cohort.

In‐house R‐scripts were developed to generate three heatmaps for each of the three cohorts. These plots represent the association analyses (SNPs*diagnosis, NIMs*diagnosis), see Figure 1 and Table S2.

HC cohort, 46 cortical thickness measures, 4 volumes, 3 pcts (*the proportion of White to Gray Matter – a proxy measure of cortical thickness*), and 1 Gaussian‐curvature (gauscurve) measure among the 200 NIMs were significantly associated with 9 markers among the 29 GMs at the level of *p* < 0.01. There were 9 ROIs (7 thicknesses, 1 volume, and 1 gauscurve) which were associated with 6 GMs at the level of *p* < 0.001, Table 3A.

**TABLE 3:** GM‐NIM associations for the HC, MCI, and AD cohorts.

SNP	Reference	Assoc. direction	Associated covariates
**(A) GM‐NIM associations for the HC cohort**			
X6.47432637	rs9381563, *CD2AP*	+	rh_S_oc.temp_lat_thickness (*p* = 0.0004) rh_S_temporal_sup_thickness (*p* = 0.0009) lh_G.S_cingul.Mid.Post_thickness (*p* = 0.0010)
X7.143108158	rs11763230, *EPHA1*	+	rh_G.S_occipital_inf_volume (*p* = 0.0010)
X11.59958380	rs2081545, *MS4A5A*	−	rh_G_occipital_middle_thickness (*p* < 0.0004)
X17.56409089	rs2632516, *BZRAP1‐AS1*	+	lh_Pole_temporal_gauscurve (*p* = 0.0010)
X18.29088958	rs8093731, *SUZ12P1*	+	rh_G.S_cingul.Mid.Post_thickness (*p* = 0.0002) rh_Lat_Fis.post_thickness (*p* = 0.0003) rh_G_occipital_middle_thickness (*p* = 0.0010)
X20.54998544	rs6014724, *CASS4*	+	rh_G.S_occipital_inf_volume (*p* = 0.0010)
**(B) GM‐NIM associations for the MCI cohort**			
X18.29088958	rs8093731, *SUZ12P1*	+	lh_MeanThickness (*p* = 0.0001) rh_MeanThickness (*p* = 0.0001) rh_S_temporal_sup_thickenss (*p* = 0.0001) lh_G_pariet_inf.Supramar_thickness (*p* = 0.0001) lh_G_temp_sup.Plan_tempo_thickness (*p* = 0.0001) rh_G_occipital_middle_thickness (*p* = 0.0001) lh_G_occipital_middle_thickness (*p* = 0.0001) rh_S_precentral.sup.part_thickness (*p* = 0.0001)
X19.1039323	rs111278892, *ABCA7*	+	lh_S_oc.temp_med.Lingual_thickness (*p* = 0.0001)
X20.54998544	rs6014724, *CASS4*	+	rh_S_oc.temp_med.Lingual_volume (*p* = 0.0001)
**(C) GM‐NIM associations for the AD cohort**			
X10.11717397	rs11257238, *ECHDC3*	+	rh_S_precentral.inf. part_thickness (*p* = 0.0009)
X18.29088958	rs8093731, *SUZ12P1*	−	3rd.Ventricle_volume (*p* = 0.0007)
X20.54998544	rs6014724, *CASS4*	+	rh_bankssts_pct (*p* = 0.0009)

Abbreviations: AD, Alzheimer's disease; HC, healthy controls; MCI, mild cognitive impairment; MMSE, Mini‐Mental State Examination; SNP, single nucleotide polymorphism.

Additional details are provided in Figure 1A and Table S2A.

MCI cohort, 116 thickness measures, 37 volumes, 22 pcts, 1 area, and 1 folding index (*foldind*) among 200 NIMs were significantly associated with the 6 markers among the 29 GMs at the level of ‘*p* < 0.01’. There were 43 ROI measures (31 thicknesses, 11 volume, and 1 pct) which were associated with 5 GMs at the level of ‘*p* < 0.001’. There were 10 ROIs (9 thicknesses and 1 volume) which were associated with 3 GMs at the level of ‘*p* < 0.0001’, Table 3B.

Additional details are provided in Figure 1B and Table S2B.

AD cohort, 23 thickness measures, 14 volumes, 15 pcts, 1 area, 1 gauscurve, and white matter hyperintensity (WMHI) among 200 NIMs were significantly associated with the 11 markers among the 29 GMs at the level of ‘*p* < 0.01’. There were three ROIs (1 thickness, 1 volume, and 1 pct) which were associated with three GMs at the level of ‘*p* < 0.001’, Table 3C.

Additional details are provided in Figure 1C and Table S2C.

### 3.4 Network analysis

**FIGURE 2:**
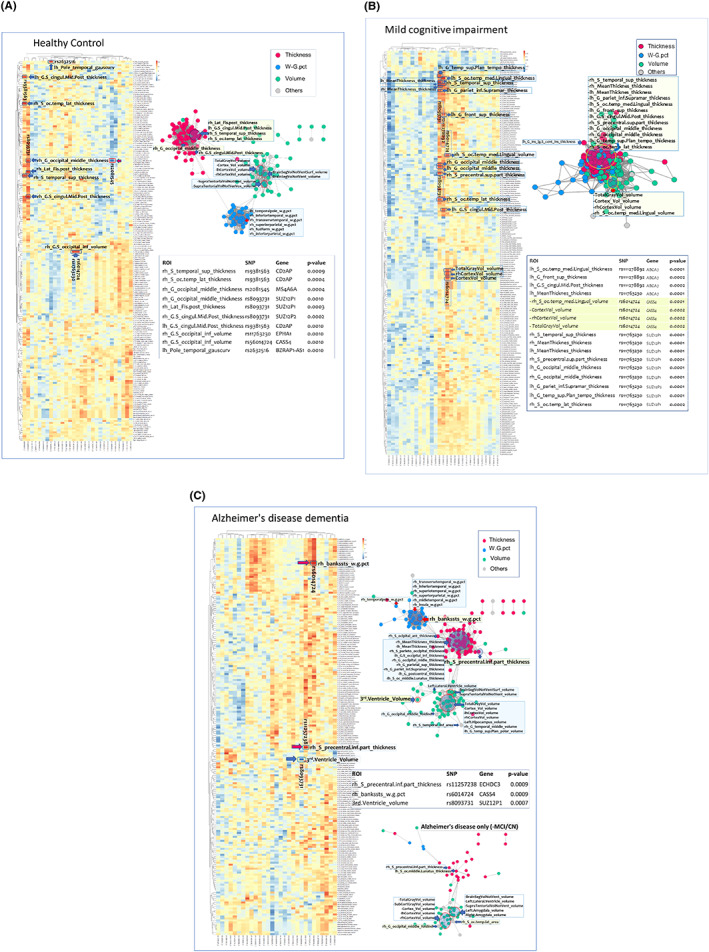
Network analysis for (A) healthy controls (HC), (B) Mild Cognitive Impairment (MCI), and (C) Alzheimer's disease (AD).

The interactions between the 29 genes and the 200 NIMs were also explored using network analysis for each of the three cohorts, HC, MCI and AD. We did not find strong network patterns solely within the 29 genes themselves, see Figure 2. However, we found strong networking patterns within the HC group. Three types of NIMs were divided into thickness, volume, and proportion of white to gray matter (pct). These three measurement groups were networking separately under the control of the 29 genes in the HC group, regardless of the *p*‐values in association analysis which is described earlier in the association analysis section.

In thickness measure group, these NIMs (mainly lh_G_precentral, rh_S_precentral.inf.part, rh_S_precentral.sup.part, rh_S_temporal_sup, both_G_precuneus, rh_G_cingul.Post.dorsal, rh_S_cingul.Marginalis, and rh_G.S_cingul.Mid.Post) were networking according to the involvement of the 29 genes.

In the volume measure group, these NIMs (mainly TotalGrayVol, CortexVol, lhCortexVol_volume, rhCortexVol, BrainSegVolNotVent, BrainSegVolNotVentSurf, rh_S_subparietal, SupraTentorialVolNotVentVox, SupraTentorialVolNotVent, SupraTentorialVolNotVentVox, rh_S_temporal_sup, and rh_G_temporal_middle) were networking according to the involvement of the 29 genes.

In the pct measure group, these NIMs (mainly rh_inferiortemporal_w.g., rh_transversetemp_w.g., rh_isthmuscingulate_w.g., rh_inferiorparietal_w.g., and rh_fusiform_w.g.) were networking according to the involvement of the 29 genes, see Figure 2A.

In the MCI cohort, the 200 NIMs were not divided as in the HC subjects. All the NIMs intermingled without a clear subgroup clustering. These three measurement groups were networking diffusely without any grouping under the control of the 29 genes, regardless of the *p*‐values in association analysis, which was described earlier in the association analysis section, see Figure 2B.

In the AD cohort, we found some patterns between the 29 genes and the 200 NIMs. Three types of NIMs were divided into thickness, volume, and pct.

These three measurement groups independently tracked neuroimaging‐genetic associations for the 29 genes in the AD subjects. The protocol for estimating the corresponding association *p*‐values was described earlier in the association analysis section.

In the thickness measure group, these NIMs (mainly both_MeanThickness, rh_S_parieto_occipital, lh_G.S_ocipital_inf, rh_G_ocipital_middle, rh_G_parietal_sup, rh_G_pariet_inf.Supramar, lh_G_postcentral, and lh_S_oc_middle.Lunatus) were involved in networks associated with the 29 SNPs.

In the volume measure group, several NIMs (mainly TotalGrayVol, CortexVol, lh/rhCortexVol, Left.Hipocampus, rh_G_temporal_middle, lh_G_temp_sup.Plan_polar, BrainSegVolNotVentSurf, and SupraTentorialVolNotVentVox) were associated with the 29 genes.

In the pct measure group, a network of NIMs (mainly rh_transversetemporal_w.g., rh_inferiortemporal_w.g., rh_superiortemporal_w.g., rh_middletemporal_w.g., lh_bankssts_w.g., and rh_insula_w.g.) were associated with the 29 genes, see Figure 2C.

### 3.5 Statistically significant ORs: MCI/HC vs. AD/HC

Figures S1 and S2 show the NIMs and GMs which discriminated between AD and HC and between MCI and HC. The values of the “OR” and “RR”, >1 or <1, identify specific biomarkers (NIM or GM) whose increase or decrease affect the risk of MCI or AD dementia, relative to the asymptomatic HC volunteers.

The AD RR of Rh_G_occipital_middle_foldind is 0.9924 in AD RR depiction, but it is a rather higher point of 1.0136 in MCI RR depiction, see Figures S1 and S2. The AD RRs of both left and right (lh/rh) parahippocampal_wg.pct, as well as (lh/rh)_ entorhinal_wg.pct, lh_middletemporal_wg.pct, lh_inferiotemporal_wg.pct, and rh_G_occipital_middle_foldind were 0.9850/0.9882, 0.9887/0.9916, 0.9924, 0.9919, and 0.9924 in AD_RR depiction, see Figure S1. In addition, the MCI RRs of (lh/rh) temporal pole_wg.pct and (lh/rh)_entorhinal_wg.pct, and rh_G_occipital_middle_foldind was 0.9902/0.9871, 0.9923/0.9880, and 1.014 in MCI RR depiction. The RRs for MCI of both (lh/rh)_parahippocampal_wg.pct were 1.0036/0.9957. Thickness and volume measures were not associated with MCI, relative to HC, see Figure S2.

In this model, the 29 SNPs did not appear to be significantly associated with AD, relative to HC, see Figure S1. The RRs for MCI of X7.143108158_T (*EPHA1*) and X19.1039323_C (*ABCA7*) were 0.9964 and 0.9973, see Figure S2.

The observed accuracy of the multinomial model (AD/HC OR; multinomial linear model of diagnosis) was 0.62 (the expectation for a null model would be 0.33) with a 95% CI of (0.6025, 0.6371), corresponding to a *p*‐value [Acc > NIR] < 2.2 × 10^−16^ and Kappa = 0.3978. Figure S3 shows us predicted diagnosis depiction.

## 4 DISCUSSION

### 4.1 SNP selection

There are two options for identifying GMs associated with AD. The first one involves GWAS analysis on the ADNI data, which includes a relatively small sample compared to other GWASs with sample sizes of tens of thousands. Another approach is to narrow the scope by utilizing the top ranked SNPs reported in various online databases, e.g., IGAP (http://web.pasteur‐lille.fr/en/recherche/u744/igap/igap_download.php), which includes summary results data from the largest (70 K samples) GWAS in AD to date. This study utilized the second approach to identify the SNPs from existing databases like GWAS in IGAP, which is based on a much larger sample compared to ADNI (IGAP ~70,000 vs. ADNI ~1200). This enormous variation between sample sizes leads to differences in statistical power to detect effects of interest, i.e., identify SNPs highly associated in NIMs and/or specific clinical outcomes. In addition, IGAP results have been extensively validated and are widely assumed to be highly reliable.

Furthermore, Jansen et al. identified the 29 SNPs extracted by meta‐analysis using PCG‐ALZ, ADSP, UKB, and deCODE as well as IGAP. We used already identified 29 SNPs by Jansen et al.^8^ Genotype dosage data for AD‐associated SNPs were extracted from the imputed ADNI genetics dosage data using sample‐major additive coding. Such 29 SNPs were selected, representing a subset of independent SNPs found to be associated with AD in the recent AD meta‐GWAS study.

### 4.2 Neuroimaging‐genetics association (prior to network analysis)

Several prior neuroimaging–genetics GWAS studies analyzed and reported specific NIMs as QTs.^5^, ^11^, ^13^ There are some similarities with our study, however previous reports do not employ network analysis.

In our HC cohort, *CD2AP* (rs9381563), *MS4A6A* (rs2081545) and *SUZ12P1* (rs8093731) were associated with thickness measures, and *EPHA1* (rs11763230) and *CASS4* (rs6014724) were associated with volume measures, especially in the occipital lobe. In general, however, cortical thickness measures were more significantly associated with the GMs compared to volume measures.

*CD2AP* (CD2 [cluster of differentiation 2] associated protein) loss of function is linked to enhanced Aβ metabolism, tau‐induced neurotoxicity, abnormal neurite structure modulation and reduced blood–brain barrier integrity.^49^ *CD2AP* is expressed in both neuron and microglia in the brain and postulated to be involved in immune system regulation.^46^ To the best of our knowledge, this is the first report of neuroimaging association of *CD2AP* gene to date. *MS4A6A* (membrane‐spanning 4‐domains, subfamily A, member 6A) has been identified as susceptibility loci of AD by several recent GWAS, whereas little is known about the potential roles of these variants in the brain structure of AD. While *MS4A6A* genotype was strongly positively related to atrophy rate in the left middle temporal, precuneus and entorhinal on MRI in the MCI subgroup in a study,^50^ in our study, this GM was mainly negatively influencing cortical thickness in the right middle occipital gyrus in the HC. Although the exact mechanisms underlying the effects of *MS4A6A* on AD pathogenesis are still largely unknown, the role of *MS4A6A* in AD progression might be mediated by modifying neuroimaging changes, possibly by affecting immune system function.^51^, ^52^ *SUZ12P1* (SUZ12 [suppressor of zeste 12] pseudogene 1) is a pseudogene, which is a long noncoding RNA promoting the proliferation of and inhibiting apoptosis of prostate cancer.^53^ To our knowledge, the *SUZ12P1* gene is not known to be associated with NIMs and this is the first report of such *SUZ12P1*‐neuroimaging association. *EPHA1* (ephrin type‐A receptor 1) plays a role in immunity and endocytosis and regulates cell morphology and motility, including permeability of the blood–brain barrier to leucocytes.^54^ *EPHA1* is known to be highly expressed in cerebral cortex and hippocampus and plays a crucial role in cortical and axonal development. Wang et al. reported that AD patients with an allele of *EPHA1* (A allele) having enhanced rate of cerebral glucose metabolism in the right lateral occipitotemporal gyrus may not have hippocampal atrophy. These authors suggested that in AD, *EPHA1* expression can play a protective role,^55^ even though in our study, we could not confirm this protective role. *CASS4* (Cas scaffolding protein family member 4) regulates focal adhesion integrity and cell spreading and plays a role in cytoskeleton and axon development and tau metabolism, affecting cellular adhesion, migration and motility.^54^

In the MCI cohort, *SUZ12P1* (rs8093731) was very significantly associated with a number of cortical thickness measures, and *CASS4* (rs6014724) was associated with volume measures. Cortical thickness and volume were significantly associated with various GMs, however, the statistical significance of thickness metrics was more profound. *SUZ12P1* was positively associated with some thickness measures in the HC cohort. There was some evidence that *SUZ12P1* may be associated with some thickness measures, which may facilitate tracking the transition from HC to the MCI stage. Similarly to the findings in the HC cohort, *CASS4* was positively associated with a volume measure in the occipital region. It is possible that *CASS4* may also be associated with volume measures in the occipital area and may be useful in forecasting transition from HC to the MCI stage. On the other hand, *ABCA7* (rs111278892) was positively associated with a thickness measure of the occipital area. Associations of *ABCA7* (ATP‐binding cassette sub‐family A member 7) with both brain atrophy^56^ and amyloidosis, especially in the MCI stage, have been previously reported.^57^, ^58^ *ABCA7* may influence AD risk via cholesterol transfer to *APOE* or by clearing Aβ aggregates.^59^, ^60^ The ABCA7 gene is strongly expressed in the hippocampus subfield CA1^61^ and showed a significant association with hippocampal atrophy^56^ and gray matter density.^62^ Like this, *ABCA7* is quite well known gene that is associated with some NIMs in AD.

In the AD cohort, some metrics of thickness, pct and volume (3rd ventricle) were significantly associated with the GMs. *ECHDC3* (rs11257238) was significantly associated with a thickness measure. The *ECHDC3* (enoyl CoA hydratase domain containing 3) is known to be involved in fatty acid biosynthesis in mitochondria.^63^ Desikan et al. reported that the gene expression of *ECHDC3* was changed in opposite directions in the AD cohort,^64^ and in our study, the gene was significantly associated with a thickness measure in the AD cohort. This gene may need to be further investigated in the future neuroimaging genetics studies. The *CASS4* was significantly associated with a pct measure in the AD cohort, which was different from the HC and MCI cohorts. The *SUZ12P1* was significantly associated with the 3rd ventricle, however, ventricular volume may also inversely reflect cortical volume changes. Finally, *SUZ12P1* and *CASS4* were significantly and differentially associated with some ROIs in the HC, MCI, and AD cohorts.

Cortical thickness is a signature marker for memory functioning across the adult lifespan. Among asymptomatic healthy individuals, the degree of cortical thinning predicts progression to clinical AD.^65^, ^66^ Moreover, our study suggests that cortical thickness may be an important measure of early detection of cognitive impairment progressing from HC to MCi and eventually leading to AD pathogenesis.^67^, ^68^, ^69^ Previous findings suggest that (1) cortical thickness and cortical surface area are independent, both globally and regionally; and (2) gray matter volume is tracked by both metrics, even though cortical thickness is less influential than surface area.^70^, ^71^ We found meaningful associations between 200 NIMs and 29 GMs for cortical thickness and regional ROI volume measures. However, cortical surface area appears as a less sensitive measure in individualized analysis within each cohort. Further studies are necessary to determine the intricate relationships between regional morphometry metrics, such as cortical volume, surface area, and thickness, specific genotypic markers, such as SNPs, and different clinical phenotypes.

On the other hand, our research finding suggests that cortical thickness may represent an important factor for tracking and discriminating subtle differences between HC and MCI cohorts. Yet, the importance of this association may not yet be extended to the AD cohort.^5^ Our current research provides evidence that cortical thickness measures are important early on, prior to dementia onset, but their importance may taper off after dementia diagnosis.

All 29 GMs reported by Jansen, et al.^8^ were found to play a crucial role in the immune system. Biological implications potentiate the hypothesis that AD pathogenesis involves an interplay between inflammation and lipids, as lipid changes might harm immune responses of microglia and astrocytes, and vascular health of the brain.^72^ Whereas, our findings indicate that during the very early phases of the HC‐MCI‐AD progression, the disease pathogenesis may be detected by accurate measurements of cortical thickness change. Based on this assumption, cortical thickness changes might be detected initially as subtle brain changes before other measurements, such as volume, pcts, surface area, folding index, gauscurve, etc. In the AD pathogenesis, most of these 29 GMs seem to be functioning in immune and lipid systems. However, we found that the associations between the genetic and the 200 NIMs are rather subtle and we suspect that the 29 GMs can affect specific neuroimaging changes that may be mediated by the broader functioning of immune and lipid systems. Delineating the precise molecular mechanisms linking ‘genomic traits’ with ‘cognitive deficits’ via ‘immune system dysfunction’ may reveal underlying mechanistic effects manifesting as brain anatomical changes (NIMs), genetic phenotypes (SNPs), and specific clinical outcomes (pathological states). Some evidence for this immune and lipid system hypothesis was recently reported.^72^, ^73^

### 4.3 Network analysis

To obtain the most reliable networks of 200 NIMs, threshold values for the network prediction were carefully chosen to meet the scale‐free topology criterion as used in other recent studies based on biological network analysis.^74^, ^75^, ^76^ No specific network patterns were identified jointly within all the 29 genes, which was contrary to our initial expectations. Hence, we performed network analyses using individual GMs.

We found specific network patterns within the respective HC and AD groups. The NIMs were divided into three thickness, volume and pct metrics under the control of 29 GMs. In the MCI cohort, transient intermingled stages without the patterning were traced and compared with the HC and AD cohorts. We found some specific AD‐proper networking patterns (HC and MCI patterns were subtracted) as well between the GMs and the NIMs. In general, the two types of NIMs were divided into thickness and volume measures. Pct measures were not highly impactful in this AD‐proper networking, Figure 2D. This may imply that in the demented AD cohort, pct measures are not homogeneously tracking the observed disease pathogenesis.

### 4.4 Statistically significant ORs and RRs in MCI/HC and AD/HC comparisons

We also calculated the ORs to study the relationships of AD and MCI to HC in terms of AD pathogenesis. In AD RR, rh_G_occipital_middle_foldind (cortical folding index) is protective, but in MCI RR, it appears detrimental rather than protective. The folding index is a specific morphometric measure of cortical integrity. It complements other measures like surface area, volume, and cortical thickness. The folding index captures the regional cortical curvature patterns. We hypothesize that the folding index might play an inhibiting role (detrimental factor) for changing from HC to MCI, but its impact reverses (as a protective factor) for changing from MCI to AD dementia. Higher folding index values correspond to highly convolved surface folding, and lower index values correspond to flatter cortical surface patches.

In MCI RR, left and right parahippocampal_wg.pct are involved as a risk factor and as a protective factor individually at the same time. Interestingly, rh_G_occipital_middle_thickness was negatively associated with rs2082545 (*MS4A6A*) in the HC cohort of our association analysis. This suggests that we need to pay attention to the occipital division as a NIM in terms of AD pathogenesis. Compared to the pct measures, thickness and volume measures did not appear as protective or detrimental factors, none of the 29 SNPs were highly associated with HC to AD transition. The genetic factors were less impactful, compared to the NIMs. This is not necessarily a problem as it's clear that single genes, or single SNPs, may not explain complex neurodegenerative phenomena such as memory loss, cognitive impairment, or clinical dementia. We aimed to identify statistically significant associations between regional, diagnostic, and genetic effects, using multinomial linear modeling and network analysis.

Basically, we suggest that similar genetic and epigenetic mechanisms continue to impact the structure and function of the brain throughout life. Early on, both genetics and experience guide neocortical and brain patterning, and these mechanisms continue to impact the maintenance of cortical areas and their boundaries as well as physiological area function throughout adulthood. Late in life, similar genetic mechanisms may be involved in the breakdown of brain microstructure as in early development, can either advance or ameliorate the deleterious effects of aging.^5^ These ideas can be reflected in the pathogenesis of AD as well.

### 4.5 Limitations and future directions

Potential limitations of this study reflect the relatively small sample size to analyze genetic influence on NIMs. The ADNI sample was not collected under a perfect epidemiological ascertainment strategy and the sample size was relatively small for a GWAS study, which may affect the generalizability of the findings. Because of our restricted statistical power, we were forced to constrain our analysis to SNPs that have been previously reported in Janssen et al. We used 29 SNPs from the Janssen et al. that are derived from the other diagnosis system from ADNI research subjects, which may also affect the generalizability of the findings.

For the neuroimaging genetics study, we used imputation tools to unify several separated ADNI data and to increase as much as possible the sample size of the genetic and neuroimaging ADNI data. This allowed us to aggregate the ADNI data and generate computable multimodal data objects including homogenous NIMs and GMs.

We did not manually inspect the brain scans of all participants (this is done by ADNI QC), to avoid potential subjective rater bias for location, size, or etiology of MRI‐evident infarcts in the QC protocol. So, there is a potential that minor WMHI effects may play a role in our analyses.

The sample only contained mild AD patients (CDR = 1), a relatively narrow range of illness, and is thus not fully representative of the HC‐MCI‐AD spectrum. At this point in time, ADNI does not collect gene expression/RNAseq data, and we could not complete a full network analysis in terms of neuroimaging genetics due to lack of available data.

Despite these challenges, the results are encouraging, and the proposed analytic framework appears to have a potential for enabling the discovery and localization of phenotypic imaging‐genetics associations. We believe that imaging‐genetics techniques offer important clues for the formulation of advanced methods of early detection, monitoring, and treatment of dementia.

## 5 CONCLUSIONS

Structural brain changes are important indicators of progressive memory decline, cognitive impairment, and dementia progression. Network analysis pairing morphometric NIMs with genetic indicators allows investigation of clinical and phenotypic associations that facilitate deep systematic understanding of dementia pathogenesis. This neuroimaging‐genetics study provides valuable clues to dementia onset and the prospective pathogenic trajectory. Our results are promising for untangling the intricate interrelations between brain anatomy and genetic phenotypes. Network analysis using neuroimaging measures and genotypic biomarkers provides cues to the structure of various deep brain‐networks and assists with interpreting structural imaging‐genomics association with disease. Further studies are necessary to reveal any specific mechanistic associations between GMs and NIMs and discover triggers or buffers of complex AD pathogenic traits.

## CONFLICT OF INTEREST

The authors declare that this study has no conflicts of interest.

6

## Supporting information


Appendix S1.

## Data Availability

The heterogeneous data types used in this study, which supported the reported research findings, are openly available from the archive of the Alzheimer's disease Neuroimaging Initiative (ADNI) (adni.loni.usc.edu).
