# Supplementary material for: Brain structure and allelic associations in Alzheimer's disease
Source: CNS Neurosci Ther. 2022 Dec 27;29(4):1034–48. doi: 10.1111/cns.14073 (PMC10018103; doi:10.1111/cns.14073)
Supplement: Supplementary file 1 — Appendix S1. [file CNS-29-1034-s001.docx]

$>0.5\%$**[Supplementary Method 1]**

**Neuroimaging-Genetics Association Analytics Protocol**

**Input data:**

1. **NIM**=neuroimaging measures, FS.stats.imaging.markers.associated.with.ADdx.CEU
2. FreeSurfer-extracted imaging markers’ metrics. Dim=1026(subjects)*206(NI biomarkers)
3. **GM**=genomic measures, genetic.markers.associated.with.ADdx.CEU,Dim=1026(subjects)*29(SNPs)
4. **roNIM**=rank-ordered NIM by p-values of AD association,imaging.markers.associated.with.ADdx.ANOVA

**Pre-processing:**

1. Extract the top 29 most significant NIM (based on NIM + roNIM), top29NIM
2. Compute the kernel density estimation (kde) for each top-29 neuroimaging biomarkers
3. Transform the raw top29NIM (i=1026, k=29) values (each has a different distribution) into 0, 1, 2 values using quantile function, which is estimated from the KDE density:
4. catTop29NIM(i,k)=ifelse(top29NIM(i,k)<quantile(0.33,k), 0, if else (top29NIM(i, k) > quantile(0.66, k), 2, 1))
5. catTop29NIM(i, k) values are 0 (bottom 1/3 quantile), 1 (mid-quantile), or 2 (top-1/3 quantile).

**Analytics:**

1. Now both arrays catTop29NIM(i=1026, k=29) and GM(i=1026, k=29) are congruent and have values 0, 1, 2.
2. Approach 1: Each pairs of indices (1<=m(NIM)<=29, 1<=n(GM)<=29) corresponds to a 3*3 matrix

|  | | **GM (Genetics markers)** | | | **Total** |
| --- | --- | --- | --- | --- | --- |
|  |  | 0 | 1 | 2 |  |
| **NIM (Neuroimaging Markers)** | 0 | V(m,n)(0,0) | … | … |  |
|  | 1 | … | … | … |  |
|  | 2 | … | … | V(m,n)(2,2) |  |
| **Total** | |  |  |  | V(m,n)=Sum(V(m,n)(.,.)) |

Then, compute the correlation as trace(3*3 matrix)/V(m,n).

1. -Approach 2: Each pairs of indices (1<=m(NIM)<=29, 1<=n(GM)<=29), just find directly the correlation (across subjects) between the 0|1|2 values of all the corresponding pairs of \rho(catTop29NIM( . , NIM=m), GM( . , n)).
2. -Having the 29*29 NIG-GM correlation matrix,

Generate a heatmap with dendograms (ala DOI 10.3389/fninf.2014.00041).

Generate Bubble plots (ala DOI 10.4306/pi.2015.12.1.125).

Generate circus plots.

| **No** | **Top200 Neuroimaging Markers** | **No** | **Top200 Neuroimaging Markers** | **No.** | **Top200 Neuroimaging Markers** | **No.** | **Top200 Neuroimaging Markers** |
| --- | --- | --- | --- | --- | --- | --- | --- |
| 1 | Right.Hippocampus_volume | 51 | rh_S_temporal_sup_volume | 101 | lh_S_interm_prim.Jensen_thickness | 151 | lh_precuneus_w.g.pct |
| 2 | Left.Hippocampus_volume | 52 | lh_G_precuneus_thickness | 102 | SubCortGrayVol_volume | 152 | lh_G_temp_sup.Plan_tempo_volume |
| 3 | Left.Amygdala_volume | 53 | rh_G_temp_sup.Lateral_thickness | 103 | lh_S_collat_transv_ant_thickness | 153 | rh_S_oc.temp_lat_area |
| 4 | lh_S_temporal_sup_thickness | 54 | lh_G_temp_sup.Plan_polar_thickness | 104 | lh_S_postcentral_thickness | 154 | rh_superiortemporal_w.g.pct |
| 5 | lh_G_temporal_middle_thickness | 55 | lh_S_oc.temp_lat_thickness | 105 | rh_Pole_temporal_volume | 155 | lh_S_temporal_inf_area |
| 6 | lh_G_oc.temp_med.Parahip_thickness | 56 | rh_S_oc.temp_lat_thickness | 106 | lh_S_circular_insula_sup_thickness | 156 | rh_S_cingul.Marginalis_thickness |
| 7 | Right.Amygdala_volume | 57 | lh_G_temp_sup.Plan_tempo_thickness | 107 | lh_G_oc.temp_lat.fusifor_volume | 157 | lh_G.S_transv_frontopol_volume |
| 8 | rh_S_temporal_sup_thickness | 58 | rh_entorhinal_w.g.pct | 108 | lh_S_intrapariet.P_trans_volume | 158 | rh_S_front_inf_thickness |
| 9 | lh_S_temporal_inf_thickness | 59 | lh_S_collat_transv_ant_volume | 109 | rh_S_collat_transv_ant_thickness | 159 | rh_G.S_transv_frontopol_thickness |
| 10 | rh_G_temporal_middle_thickness | 60 | rhCortexVol_volume | 110 | rh_G_precuneus_volume | 160 | lh_G_Ins_lg.S_cent_ins_thickness |
| 11 | lh_MeanThickness_thickness | 61 | rh_G_front_middle_thickness | 111 | lh_Pole_temporal_volume | 161 | rh_G.S_occipital_inf_volume |
| 12 | lh_G_temporal_inf_thickness | 62 | rh_G_precuneus_thickness | 112 | lh_S_front_middle_thickness | 162 | lh_supramarginal_w.g.pct |
| 13 | Left.Inf.Lat.Vent_volume | 63 | lh_S_subparietal_thickness | 113 | lh_S_parieto_occipital_thickness | 163 | lh_S_oc_sup.transversal_thickness |
| 14 | rh_MeanThickness_thickness | 64 | rh_G_pariet_inf.Angular_volume | 114 | rh_G.S_cingul.Mid.Post_thickness | 164 | rh_S_precentral.sup.part_thickness |
| 15 | rh_G_pariet_inf.Angular_thickness | 65 | rh_S_intrapariet.P_trans_thickness | 115 | rh_S_collat_transv_ant_volume | 165 | SupraTentorialVolNotVentVox_volume |
| 16 | Right.Inf.Lat.Vent_volume | 66 | rh_S_circular_insula_inf_thickness | 116 | lh_fusiform_w.g.pct | 166 | lh_S_collat_transv_ant_area |
| 17 | lh_G_pariet_inf.Angular_thickness | 67 | lh_middletemporal_w.g.pct | 117 | lh_S_front_inf_thickness | 167 | wm.lh.parahippocampal_volume |
| 18 | lh_G_temporal_middle_volume | 68 | TotalGrayVol_volume | 118 | rh_G_temp_sup.Lateral_volume | 168 | CSF_volume |
| 19 | rh_S_temporal_inf_thickness | 69 | rh_S_interm_prim.Jensen_thickness | 119 | rh_S_temporal_inf_area | 169 | lh_S_orbital_med.olfact_thickness |
| 20 | rh_G_temporal_inf_thickness | 70 | lh_S_circular_insula_inf_thickness | 120 | lh_bankssts_w.g.pct | 170 | SupraTentorialVolNotVent_volume |
| 21 | rh_G_oc.temp_med.Parahip_thickness | 71 | rh_G_front_sup_thickness | 121 | lh_Lat_Fis.post_thickness | 171 | wm.lh.entorhinal_volume |
| 22 | lh_S_oc.temp_med.Lingual_thickness | 72 | rh_temporalpole_w.g.pct | 122 | lh_inferiorparietal_w.g.pct | 172 | lh_S_oc_middle.Lunatus_thickness |
| 23 | lh_G_cingul.Post.dorsal_thickness | 73 | rh_G_occipital_middle_thickness | 123 | rh_bankssts_w.g.pct | 173 | rh_S_temporal_transverse_thickness |
| 24 | lh_G_oc.temp_med.Parahip_volume | 74 | lh_S_precentral.inf.part_thickness | 124 | lh_S_oc.temp_med.Lingual_volume | 174 | lh_G.S_cingul.Mid.Post_thickness |
| 25 | lh_G_oc.temp_lat.fusifor_thickness | 75 | rh_G_pariet_inf.Supramar_thickness | 125 | rh_precuneus_w.g.pct | 175 | rh_S_orbital_med.olfact_thickness |
| 26 | lh_S_temporal_inf_volume | 76 | rh_S_parieto_occipital_thickness | 126 | Left.Lateral.Ventricle_volume | 176 | rh_posteriorcingulate_w.g.pct |
| 27 | lh_S_intrapariet.P_trans_thickness | 77 | lh_G_front_sup_thickness | 127 | rh_G_cingul.Post.dorsal_volume | 177 | rh_S_subparietal_volume |
| 28 | lh_S_temporal_sup_volume | 78 | lh_G_cingul.Post.ventral_thickness | 128 | wm.rh.parahippocampal_volume | 178 | lh_caudalmiddlefrontal_w.g.pct |
| 29 | lh_entorhinal_w.g.pct | 79 | lh_temporalpole_w.g.pct | 129 | lh_Pole_temporal_gauscurv | 179 | rh_caudalmiddlefrontal_w.g.pct |
| 30 | rh_G_temporal_middle_volume | 80 | lh_inferiortemporal_w.g.pct | 130 | rh_G_temp_sup.Plan_polar_volume | 180 | lh_G.S_occipital_inf_thickness |
| 31 | rh_S_oc.temp_med.Lingual_thickness | 81 | rh_middletemporal_w.g.pct | 131 | lh_G_temp_sup.Plan_polar_volume | 181 | lh_S_temporal_sup_area |
| 32 | rh_G_temporal_inf_volume | 82 | rh_S_oc_sup.transversal_thickness | 132 | rh_parahippocampal_w.g.pct | 182 | rh_G_occipital_middle_foldind |
| 33 | lh_G_temporal_inf_volume | 83 | rh_S_precentral.inf.part_thickness | 133 | lh_parahippocampal_w.g.pct | 183 | BrainSegVolNotVentSurf_volume |
| 34 | rh_G_cingul.Post.dorsal_thickness | 84 | rh_inferiortemporal_w.g.pct | 134 | lh_G_pariet_inf.Supramar_volume | 184 | BrainSegVolNotVent_volume |
| 35 | lh_G_temp_sup.Lateral_thickness | 85 | rh_G.S_occipital_inf_thickness | 135 | rh_G_cingul.Post.ventral_thickness | 185 | lh_G_precentral_thickness |
| 36 | rh_S_temporal_inf_volume | 86 | rh_G_oc.temp_lat.fusifor_volume | 136 | lh_superiortemporal_w.g.pct | 186 | rh_transversetemporal_w.g.pct |
| 37 | rh_Pole_temporal_thickness | 87 | rh_S_circular_insula_sup_thickness | 137 | Left.Accumbens.area_volume | 187 | lh_G_postcentral_thickness |
| 38 | lh_Pole_temporal_thickness | 88 | rh_G_temp_sup.Plan_tempo_thickness | 138 | lh_G_occipital_middle_volume | 188 | lh_G.S_transv_frontopol_thickness |
| 39 | lh_G_pariet_inf.Supramar_thickness | 89 | lh_G_temp_sup.Lateral_volume | 139 | Right.Lateral.Ventricle_volume | 189 | rh_superiorfrontal_w.g.pct |
| 40 | lh_G_pariet_inf.Angular_volume | 90 | rh_S_oc.temp_med.Lingual_volume | 140 | rh_S_postcentral_thickness | 190 | rh_S_occipital_ant_thickness |
| 41 | lhCortexVol_volume | 91 | lh_G_occipital_middle_thickness | 141 | lh_S_oc.temp_lat_volume | 191 | rh_isthmuscingulate_w.g.pct |
| 42 | lh_G_front_middle_thickness | 92 | rh_G_occipital_middle_volume | 142 | X3rd.Ventricle_volume | 192 | rh_S_temporal_inf_meancurv |
| 43 | rh_S_subparietal_thickness | 93 | rh_fusiform_w.g.pct | 143 | rh_S_front_middle_thickness | 193 | WM.hypointensities_volume |
| 44 | lh_S_front_sup_thickness | 94 | lh_S_occipital_ant_thickness | 144 | rh_Pole_temporal_gauscurv | 194 | lh_S_temporal_transverse_thickness |
| 45 | rh_S_oc.temp_lat_volume | 95 | rh_inferiorparietal_w.g.pct | 145 | rh_S_collat_transv_post_thickness | 195 | lh_parsopercularis_w.g.pct |
| 46 | rh_G_oc.temp_lat.fusifor_thickness | 96 | lh_G_precuneus_volume | 146 | rh_S_oc_middle.Lunatus_thickness | 196 | lh_S_cingul.Marginalis_thickness |
| 47 | rh_G_oc.temp_med.Parahip_volume | 97 | rh_Lat_Fis.post_thickness | 147 | rh_supramarginal_w.g.pct | 197 | rh_parsopercularis_w.g.pct |
| 48 | rh_S_front_sup_thickness | 98 | lh_G_parietal_sup_thickness | 148 | rh_G_Ins_lg.S_cent_ins_thickness | 198 | rh_insula_w.g.pct |
| 49 | rh_G_temp_sup.Plan_polar_thickness | 99 | lh_G_cingul.Post.dorsal_volume | 149 | lh_G_subcallosal_thickness | 199 | lh_G_front_middle_volume |
| 50 | CortexVol_volume | 100 | rh_G_parietal_sup_thickness | 150 | rh_S_intrapariet.P_trans_volume | 200 | rh_superiorparietal_w.g.pct |

Supplementary Table 1. The_200_Neuroimaging Markers

Supplementary Table 2. Neuroimaging-Genetics Association Analytics results

Supplementary Table 2(A). Cognitively Healthy Control cohort (HC)

| **SNP** | **Gene** | **HC** | | | | | | |
| --- | --- | --- | --- | --- | --- | --- | --- | --- |
|  |  | **p < 0.01** | | | | **p < 0.001** | | |
|  |  | **Thickness (39)** | **Volume (3)** | **Pcs (3)** | | **Thickness (7)** | **Volume (2)** | **Gauscurve (1)** |
| X6.32583357_A | *[HLA-DRB1]* | lh_S_temporal_inf, lh_G_cingul.Post.ventral, lh_Lat_Fis.post |  |  | |  |  |  |
| X6.47432637_C | *[CD2AP]* | rh_S_precentral.inf.part, rh_G.S_cingul.Mid.Post, rh_MeanThickness, rh_S_circular_insula_inf, rh_G_temporal_middle |  |  | | rh_S_temporal_sup, lh_G.S_cingul.Mid.Post, rh_S_oc.temp_lat |  |  |
| X7.143108158_T | *[EPHA1]* | rh_G.S_occipital_inf |  |  | |  | rh_G.S_occipital_inf |  |
| X11.59958380_C | *[MS4A6A]* | lh_G_temporal_middle, rh_S_temporal_sup, lh_S_temporal_inf, lh_G_pariet_inf.Angular, rh_G_temp_sup.Lateral, rh_G_front_middle, rh_G_temp_sup.Plan_tempo, rh_S_precentral.sup.part |  |  | | rh_G_occipital_middle |  |  |
| X16.31133100_G | *[KAT8]* | lh_S_front_sup, rh_G_front_sup, lh_G_front_sup, rh_G_pariet_inf.Supramar, rh_S_precentral.sup.part |  |  | |  |  |  |
| X17.56409089_G | *[BZRAP1-AS1]* |  |  | rh_fusiform_w.g, lh_fusiform, rh_insula_w.g | |  |  | lh_Pole_temporal |
| X18.29088958_C | *[SUZ12P1]* | lh_S_temporal_sup, rh_S_temporal_sup, lh_MeanThickness, rh_MeanThickness, lh_G_pariet_inf.Angular, lh_S_oc.temp_med.Lingual, rh_S_oc.temp_med.Lingual, rh_G_oc.temp_lat.fusifor, lh_G_precuneus, rh_S_circular_insula_inf, rh_S_parieto_occipital, lh_G_parietal_sup, lh_S_parieto_occipital, rh_S_precentral.sup.part, lh_S_orbital_med.olfact, rh_S_occipital_ant |  |  | | rh_G_occipital_middle, rh_G.S_cingul.Mid.Post, rh_Lat_Fis.post |  |  |
| X19.1039323_C | *[ABCA7]* | lh_G_subcallosal_thickness | Right.Amygdala, lh_G_pariet_inf.Supramar, SubCortGrayVol | |  |  |  |  |
| X20.54998544_A | *[CASS4]S* |  |  | |  |  | rh_G.S_occipital_inf |  |

Supplementary Table 2(B). Mild Cognitive Impairment (MCI) cohort

| **SNP** | **Gene** | **MCI** | | | | | | | | | |
| --- | --- | --- | --- | --- | --- | --- | --- | --- | --- | --- | --- |
|  |  | **p < 0.01** | | | | | **p < 0.001** | | | | |
|  |  | **Thickness (82)** | **Volume (24)** | | **Pcs (22)/ foldind (1)/ area(1)** | | **Thickness (34)** | **Volume (13)** | | **Pcs** | |
| X4.11026028_A | *[CLNK]* | lh_G_oc.temp_med.Parahip, lh_MeanThickness, rh_MeanThickness, lh_S_oc.temp_med.Lingual, lh_G_cingul.Post.dorsal, lh_G_oc.temp_lat.fusifor, lh_S_intrapariet.P_trans, rh_G_cingul.Post.dorsal, lh_G_temp_sup.Lateral, lh_S_front_sup, rh_S_front_sup, lh_S_subparietal, rh_S_intrapariet.P_trans, rh_G_occipital_middle, rh_S_parieto_occipital, rh_S_oc_sup.transversal, rh_Lat_Fis.post, lh_S_postcentral, lh_S_parieto_occipital, lh_S_front_inf, lh_Lat_Fis.post, lh_S_oc_sup.transversal, lh_G.S_cingul.Mid.Post, lh_G_precentral | |  | rh_G_occipital_middle_foldind (folding index) | | lh_S_temporal_sup, lh_S_circular_insula_inf |  | |  | |
| X6.32583357_A | *[HLA-DRB1]* |  | rh_S_oc.temp_lat, lh_G_oc.temp_lat.fusifor, rh_S_subparietal, SupraTentorialVolNotVentVox, SupraTentorialVolNotVent, BrainSegVolNotVentSurf, BrainSegVolNotVent | | |  |  |  | |  | |
| X8.27464929_A | *[CLU/PTK2B]* |  |  | | rh_S_temporal_ rh_S_temporal_inf _area (area) | | rh_G_parietal_sup |  | |  | |
| X18.29088958_C | *[SUZ12P1]* | rh_G_pariet_inf.Angular, lh_S_temporal_inf, lh_S_oc.temp_med.Lingual, lh_S_intrapariet.P_trans, lh_G_front_middle,_ rh_G_front_middle, rh_S_intrapariet.P_trans, rh_S_circular_insula_inf, rh_S_interm_prim.Jensen, lh_S_precentral.inf.part, rh_G_pariet_inf.Supramar, rh_S_parieto_occipital, rh_S_oc_sup.transversal, rh_G.S_occipital_inf, rh_Lat_Fis.post, lh_G_parietal_sup, lh_S_postcentral, lh_S_front_inf, lh_Lat_Fis.post, rh_G_cingul.Post.ventral, rh_G_Ins_lg.S_cent_ins, rh_S_temporal_transverse, lh_G_precentral, lh_G_postcentral, lh_G.S_transv_frontopol, rh_S_occipital_ant | rh_G.S_occipital_inf, rh_Lat_Fis.post, | | lh_G_parietal_sup, lh_S_postcentral, lh_S_front_inf, lh_Lat_Fis.post, rh_G_cingul.Post.ventral, rh_G_Ins_lg.S_cent_ins, rh_S_temporal_transverse, lh_G_precentral, lh_G_postcentral, lh_G.S_transv_frontopol, rh_S_occipital_ant | | lh_S_temporal_sup, lh_G_temporal_middle, rh_G_temporal_middle, lh_MeanThickness, rh_MeanThickness, lh_G_pariet_inf.Angular, rh_S_temporal_inf, rh_S_oc.temp_med.Lingual, lh_G_temp_sup.Lateral, , rh_G_temp_sup.Lateral, lh_G_pariet_inf.Supramar, rh_S_collat_transv_post lh_G_temp_sup.Plan_tempo, rh_G_occipital_middle, rh_S_oc.temp_lat, rh_G_temp_sup.Plan_tempo, lh_G_occipital_middle, rh_S_precentral.sup.part, | |  |  | |
| X19.1039323_C | *[ABCA7]* | rh_S_temporal_sup, rh_G_pariet_inf.Angular, rh_S_temporal_inf, rh_S_oc.temp_med.Lingual, lh_G_temp_sup.Lateral, lh_G_pariet_inf.Supramar, lh_G_front_middle, lh_S_front_sup, rh_G_oc.temp_lat.fusifor, rh_G_front_middle, lh_S_subparietal, rh_G_occipital_middle, rh_G_pariet_inf.Supramar, rh_S_parieto_occipital, rh_G_temp_sup.Plan_tempo, lh_G_occipital_middle, lh_G_parietal_sup, lh_S_postcentral, lh_S_parieto_occipital, rh_G.S_cingul.Mid.Post, lh_S_front_inf, rh_S_postcentral, rh_S_collat_transv_post, rh_S_cingul.Marginalis, lh_G_precentral, lh_S_temporal_transverse | lh_G_pariet_inf.Supramar, lh_G_front_middle, | | lh_S_front_sup, rh_G_oc.temp_lat.fusifor, rh_G_front_middle, lh_S_subparietal, rh_G_occipital_middle, rh_G_pariet_inf.Supramar, rh_S_parieto_occipital, rh_G_temp_sup.Plan_tempo, lh_G_occipital_middle, lh_G_parietal_sup, lh_S_postcentral, | | lh_S_oc.temp_med.Lingual, lh_MeanThickness, rh_MeanThickness, lh_G_cingul.Post.dorsal, lh_G_oc.temp_lat.fusifor, lh_G_precuneus, rh_G_front_sup, lh_G_front_sup, rh_G.S_occipital_inf, lh_Lat_Fis.post, lh_G.S_cingul.Mid.Post, lh_G.S_occipital_inf, lh_G_postcentral | lh_S_front_inf, rh_S_postcentral, rh_S_collat_transv_post, rh_S_cingul.Marginalis, lh_G_precentral, rh_G_precuneus | | lh_S_temporal_transverse | |
| X20.54998544_A | *[CASS4]SS4]* | lh_MeanThickness, rh_G_cingul.Post.dorsal, lh_S_circular_insula_inf, rh_S_collat_transv_post, lh_G.S_cingul.Mid.Post, lh_G_postcentral | Right.Hippocampus, Right.Amygdala, Left.Inf.Lat.Vent, rh_G_temporal_middle, lh_S_collat_transv_ant, rh_G_temp_sup.Plan_polar, lh_G_occipital_middle, lh_G_temp_sup.Plan_tempo, lh_G.S_transv_frontopol, SupraTentorialVolNotVentVox,SupraTentorialVolNotVent, BrainSegVolNotVentSurf, BrainSegVolNotVent | | |  | lh_G_precentral | rh_S_oc.temp_med.Lingual, CortexVol, lhCortexVol, rhCortexVol, TotalGrayVol, lh_G_pariet_inf.Supramar, lh_G_front_middle | | |  |

Supplementary Table 2(C). Alzheimer’s Disease Dementia (AD) cohort

| **SNP** | **Gene** | **ADD** | | | | | | | | | |
| --- | --- | --- | --- | --- | --- | --- | --- | --- | --- | --- | --- |
|  |  | **p < 0.01** | | | | | **p < 0.001** | | | | |
|  |  | **Thickness (22)** | **Volume (13)** | **Pcs (14) / WMHI** | | **Area (1)/ Gauscurve(1)** | **Thickness (1)** | **Volume(1)** | | **Pcs(1)** | |
| X2.127891427_A | *[BIN1]* | both_G_front_middle, rh_G.S_transv_frontopol |  |  | |  |  |  | |  | |
| X4.11723235_A | *[HS3ST1]* | rh_S_temporal_sup, rh_G_pariet_inf.Angular, rh_G_cingul.Post.dorsal | wm.lh.parahippocampal | rh_bankssts | |  |  |  | |  | |
| X7.99971834_A | *[ZCWPW1]* | lh_S_temporal_transverse, rh_G.S_occipital_inf |  | WMHI | |  |  |  | |  | |
| X10.11717397_T | *[ECHDC3]* | lh_S_postcentral lh_G_occipital_middle |  | rh_insula_w.g | |  | rh_S_precentral.inf. part | |  |  | |
| X11.121435587_T | *[SORL1]* | rh_G_temporal_middle |  | rh_inferiorparietal_w.g, rh_superiorparietal_w.g | |  |  |  | |  | |
| X11.59958380_C | *[MS4A6A]* | rh_G_temp_sup.Lateral, lh_G_temp_sup.Plan_tempo, lh_G_postcentral, rh_G_occipital_middle |  |  | |  |  |  | |  | |
| X11.85776544_G | *[PICALM]* | rh_Pole_temporal, rh_S_orbital_med.olfact | lh_G_temp_sup.Plan_tempo, lh_G_temp_sup.Plan_polar, rh_Pole_temporal | |  |  |  |  | |  | |
| X14.92938855_G | *[SLC24A4]* | rh_G_front_sup, lh_G_front_sup | lh_G_temporal_inf | |  |  |  |  | |  | |
| X15.59022615_T | *[ADAM10]* |  | BrainSegVolNotVentSurf, BrainSegVolNotVent, SubCortGrayVol, rh_G_oc.temp_lat.fusifor, rh_G_cingul.Post.dorsal, rh_S_temporal_inf, SupraTentorialVolNotVentVox, SupraTentorialVolNotVent | |  | rh_S_temporal_inf_area |  |  | |  | |
| X18.29088958_C | *[SUZ12P1]* |  |  |  | | rh_Pole_temporal_gauscurve |  | 3rd.Ventricle_volume | | |  |
| X20.54998544_A | *[CASS4]SS4]* | rh_S_oc.temp_med.Lingual, lh_G.S_occipital_inf, lh_S_parieto_occipital |  | both_inferiorparietal, rh_superiorparietal, rh_precuneus, both_caudalmiddlefrontal, both_supramargial, rh_superiorfrontal, lh_parsopercularis | |  |  |  | | rh_bankssts | |

Supplementary Table 3. The ROI dictionary table for the Supplementary Table 1.

| **ROI Code** | **ROI Name** | **ROI Functional and Anatomical Importance** |
| --- | --- | --- |
| **HC** (P< 0.001, for the association between NIMs and SNPs) | | |
| rh_S_temporal_sup_thickness | Cortical thickness in the right superior temporal sulcus | The superior temporal sulcus has been shown to produce strong responses when subjects perceive stimuli in research areas that include theory of mind, biological motion, faces, voices, and language. [REF=http://en.wikipedia.org/wiki/Superior_temporal_sulcus] |
| rh_S_oc.temp_lat_thickness | Cortical thickness in the right lateral occipito-temporal sulcus |  |
| rh_G_occipital_middle_thickness | Cortical thickness in the right middle occipital gyrus |  |
| rh_Lat_Fis.post_thickness | Cortical thickness in the posterior segment of the right lateral fissure(sulcus) | The lateral fissure is a deep sulcus in each hemisphere that separates the frontal and parietal lobes from the temporal lobe. [REF= https://en.wikipedia.org/wiki/Lateral_sulcus] |
| rh_G.S_cingul.Mid.Post_thickness | Cortical thickness in the middle-posterior part of the right cingulate gyrus and sulcus | It receives inputs from the thalamus and the neocortex, and projects to the entorhinal cortex via the cingulum. It is an integral part of the limbic system, which is involved with emotion formation and processing, learning, and memory. [REF=https://en.wikipedia.org/wiki/Cingulate_cortex] |
| lh_G.S_cingul.Mid.Post_thickness | Cortical thickness in the middle- posterior part of the left cingulate gyrus and sulcus | Refer to the upper part. |
| rh_G.S_occipital_Inf_volume | Volume of the right inferior occipital gyrus and sulcus |  |
| lh_Pole_temporal_gauscurv | Gauscurv of the left temporal pole | The temporal pole is mainly connected with the amygdala, the hippocampus, the superior temporal gyrus, and the occipitobasal cortex. [REF=https://pubmed.ncbi.nlm.nih.gov/12424085/] |
| **MCI** (P< 0.0002, for the association between NIMs and SNPs) | | |
| lh_S_oc.temp_med.Lingual_thickness | Cortical thickness in the lingual part of the left medial occipito-temporal sulcus |  |
| lh_G_front_sup_thickness | Cortical thickness in the left superior frontal gyrus |  |
| lh_G.S_cingul.Mid.Post_thickness | Cortical thickness in the middle-posterior part of the left cingulate gyrus and sulcus | Refer to the upper part. |
| *- rh_S_oc.temp_med.Lingual_volume* | Volume of the lingual part of the right medial occipito-temporal sulcus |  |
| *- CortexVol_volume* | Volume of the whole cortex |  |
| *- rhCortexVol_volume* | Volume of the right cortex |  |
| *- TotalGrayVol_volume* | Volume of the total gray matter |  |
| rh_S_temporal.sup_thickness | Cortical thickness in the right superior temporal sulcus | The superior temporal sulcus has been shown to produce strong responses when subjects perceive stimuli in research areas that include theory of mind, biological motion, faces, voices, and language. [REF=http://en.wikipedia.org/wiki/Superior_temporal_sulcus] |
| rh_MeanThickness_thickness | Right mean cortical thickness |  |
| lh_MeanThickness_thickness | Left mean cortical thickness |  |
| rh_S_precentral.sup.part_thickness | Cortical thickness in the right superior part of the precentral sulcus | The precentral gyrus is a prominent gyrus on the surface of the posterior frontal lobe of the brain. It is the site of the primary motor cortex that in humans is cytoarchitecturally defined as Brodmann area 4. [REF=https://en.wikipedia.org/wiki/Precentral_gyrus] |
| lh_G_occipital_middle_thickness | Cortical thickness in the left middle occipital gyrus |  |
| rh_G_occipital_middle_thickness | Cortical thickness in the right middle occipital gyrus |  |
| lh_G_temp_sup.Plan_tempo_thickness | Cortical thickness in the planum temporale (or temporal plane) of the left superior temporal gyrus | The planum temporale is the cortical area just posterior to the auditory cortex within the lateral fissure. It is a triangular region which forms the heart of Wernicke's area, one of the most important  functional areas for language. [REF=https://en.wikipedia.org/wiki/Planum_temporale] |
| rh_S_oc.temp_lat_thickness | Cortical thickness in the right lateral occipito-temporal sulcus |  |
| **AD dementia** (P< 0.001, for the association between NIMs and SNPs) | | |
| rh_S_precentral.inf.part_thickness | Cortical thickness in the inferior part of the right precentral sulcus | Refer to the upper part. |
| rh_bankssts_w.g.pct | Ratio of white matter to gray matter in the right hemisphere banks of superior temporal sulcus | Within the FreeSurfer definition, this reflects primarily the posterior aspect of the superior temporal sulcus. |
| 3rd.Ventricle_volume | Volume of the 3^rd^ ventricle |  |
| **MCI/HC and AD/HC odds-ratios** | | |
| rh_G_occipital_middle_foldind | Folding index of the right middle occipital gyrus |  |


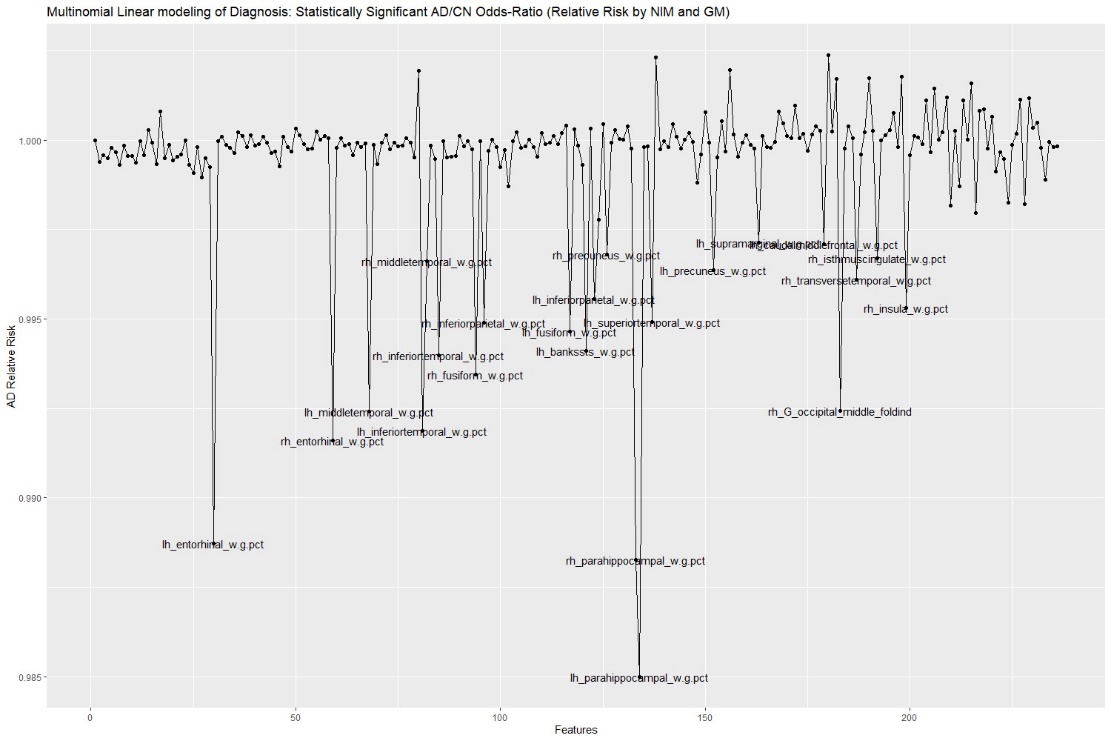


Supplementary Figure 1. Statistically significant AD/HC odds-ratio

(Multinomial linear model of diagnosis).


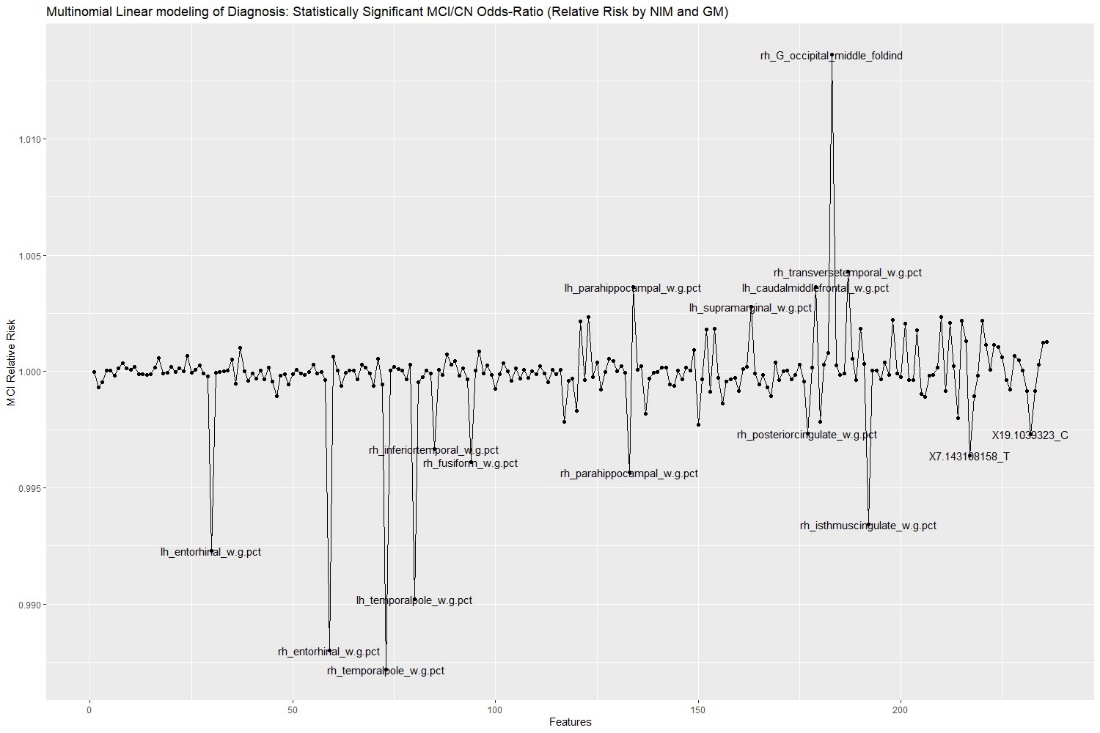


Supplementary Figure 2. Statistically significant MCI/HC odds-ratio

(Multinomial linear model of diagnosis).


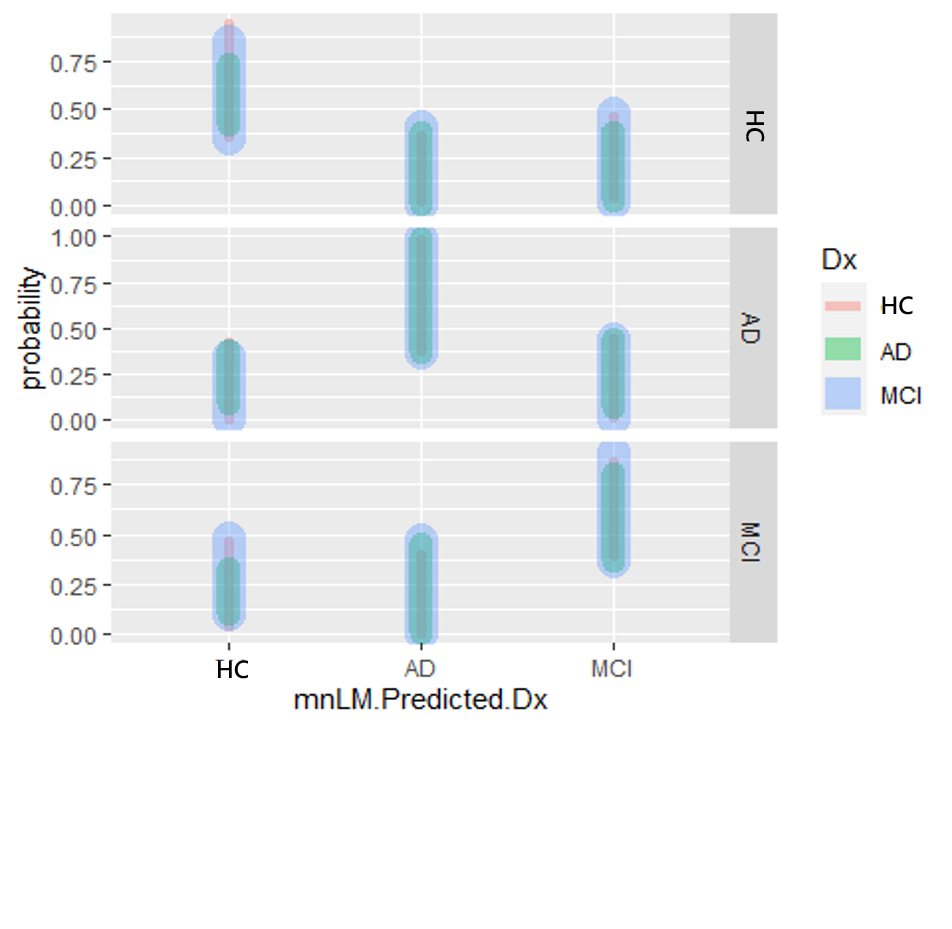


Supplementary Figure 3. Clinical vs. linear model-predicted diagnoses (Dx).

(The vertical axis shows the probabilities for each Dx class label

derived from the multinomial linear model)
